# Incidence of Keratoconus and Its Association with Systemic Comorbid Conditions: A Nationwide Cohort Study from South Korea

**DOI:** 10.1155/2020/3493614

**Published:** 2020-03-25

**Authors:** Jong Youn Moon, Jesang Lee, Yoon Hyung Park, Eun-Cheol Park, Si Hyung Lee

**Affiliations:** ^1^Institute of Health Services Research, Yonsei University, College of Medicine, Seoul, Republic of Korea; ^2^Department of Ophthalmology, Soonchunhyang University Hospital Bucheon, Bucheon, Republic of Korea; ^3^Department of Preventive Medicine, Soonchunhyang University, College of Medicine, Cheonan, Republic of Korea

## Abstract

**Purpose:**

To determine the incidence of keratoconus and to determine its possible association with common systemic diseases using a nationwide cohort.

**Methods:**

This retrospective nationwide cohort study included 1,025,340 subjects from the Korean National Health Insurance Service-National Sample Cohort database from 2004 to 2013. Estimates for incidence rates of keratoconus were identified. After 1 : 5 matching using propensity scores, associations between keratoconus and certain systemic comorbidities were determined using multivariate logistic regression analysis.

**Results:**

The incidence during the same period was 15.1 cases per 100,000 person-years. Adjusted logistic regression analysis after propensity score matching revealed significant associations between keratoconus and allergic rhinitis (odds ratio (OR): 1.86; 95% confidence interval (CI): 1.63–2.13; *p* < 0.001), asthma (OR: 1.20; 95% CI: 1.06–1.36; *p* < 0.001), atopic dermatitis (OR: 1.33; 95% CI: 1.13–1.56; *p* < 0.001), and diabetes mellitus (DM) (OR: 1.35; 95% CI: 1.15–1.58; *p* < 0.001).

**Conclusion:**

Estimates of the incidence of keratoconus may help in the planning of eye-care policies, and the results of this study determined the associations between allergic diseases and keratoconus. Conflicting results regarding the association between keratoconus and DM should be further evaluated.

## 1. Introduction

Keratoconus is a chronic, progressive, noninflammatory ectatic corneal condition that leads to decreased visual acuity because of increased myopia and irregular astigmatism. These conditions are known to affect patients 18–40 years of age. The severity of the disease varies from a subclinical stage to a severely progressive stage, resulting in corneal scarring, perforation, or even blindness [1, 2]. Treatment also varies from wearing glasses or rigid contact lenses to complex surgical procedures such as keratoplasty to restore visual acuity [1].

The previously reported annual incidence of keratoconus varied greatly from 1.3 to 32.3 cases per 100,000, according to the country where the study was conducted [3]. This wide range of reported estimates may be due to ethnic differences, differences in accessibility because of regional differences, and discordant study designs [3]. Additionally, with recent advances in refractive surgery and the development of delicate corneal topographic devices and software, keratoconus is diagnosed more frequently than before, especially in the early subclinical stages, which may also account for some disparities in the estimates of annual incidence of keratoconus [4, 5].

While the pathophysiology of keratoconus is currently not clearly known, a few previous studies investigated risk factors for keratoconus to clarify its pathophysiological mechanism [4]. The reported risk factors for keratoconus include ocular allergy, atopic dermatitis, connective tissue disorders, and Down syndrome [6–9]. Contradictory results exist regarding the role of diabetes mellitus (DM) in the development of keratoconus, with the possibility of increasing or reducing the risk of developing corneal ectasia [10–12]. Considering ethnic differences in the incidence of keratoconus, possible associations between systemic comorbidities and keratoconus should be clearly determined in patients with Asian ethnicity, and reports regarding the risk factors for keratoconus in the Republic of Korea should be clearly defined.

Based on these considerations, we evaluated the incidence of keratoconus and investigated associations between various systemic conditions and keratoconus using data from a nationwide sample of approximately 1 million residents from the National Health Insurance Service-National Sample Cohort 2002–2013 (NHIS-NSC 2002-2013).

## 2. Methods and Materials

### 2.1. Database

This study used data from the NHIS-NSC 2002–2013. The Korean National Health Insurance Service (KNHIS), which is a mandatory single medical insurer system, has been managing national health insurance in the Republic of Korea since 1989. All citizens are required to enroll in the system, and it provides nearly the entire healthcare coverage in the Republic of Korea. The KNHIS developed the NHIS-NSC for research purposes, which contains personal demographic records for all medical information related to insurance claims. The NHIS-NSC sampled 1,025,340 individuals from among 46,605,433 individuals in the NHID (2.2%) in 2002 and followed them until 2013 [13]. The study design was approved by the Institutional Review Board of Soonchunhyang University Bucheon Hospital, Gyeonggi-do, Republic of Korea. This study adhered to the tenets of the Declaration of Helsinki, and the need for written informed consent was waived.

### 2.2. Selection of Study Samples

To exclude chronic keratoconus patients, we excluded patients who were diagnosed with keratoconus in 2002 and 2003 and included patients who were diagnosed with keratoconus from 2004. The keratoconus cases were identified as patients who had the following Korean Classification of Diseases code as the main diagnosis code: H186 and H198, and had a keratometry measurement code (E6870).

Control subjects were selected from individuals who did not have keratoconus diagnosis codes, each year, using propensity scores with nearest neighbor matching in a 1 : 5 ratio to keratoconus patients [14]. Propensity scores were calculated using sociodemographic parameters, including age group (≤19, 20–59, and ≥60 years of age), sex, income (≤40^th^, 41^st^–70^th^, and ≥71^st^ percentiles), residential area (urban, metropolitan cities, cities, rural, and all other areas), and year of enrollment.

### 2.3. Incidence and Comorbidity

In the matched populations, we compared the incidence of keratoconus. The earliest date of the keratoconus diagnosis was defined as the index date and the incident time, and the patient was considered an incident case in that corresponding year. Comorbid conditions included allergic rhinitis (codes), asthma (codes), atopic dermatitis (codes), aortic dissection (codes), sleep apnea (codes), collagen vascular diseases (codes), DM (codes E10–E14), mitral valve prolapse (codes), and Down syndrome (code) that were diagnosed between 2002 and 2008. Because the purpose of this study was to investigate associations between systemic diseases and keratoconus, and not necessarily to identify causative relationships between the conditions, we included cases and controls without considering the date when the diagnosis was made during the study period.

### 2.4. Statistical Analysis

Baseline characteristics are expressed as the mean and standard deviation or number and percentage. The chi square test was used to compare groups. Multivariate logistic regression analysis was performed to calculate odds ratio (OR) with a 95% confidence interval (CI) to evaluate the potential association between keratoconus and various systemic diseases. The variables included in multivariate analyses were age, sex, income, residential area, and systemic diseases of interest that were previously reported to be associated with keratoconus. All statistical analyses were conducted using SAS software, version 9.4 (SAS Institute, Cary, NC, USA), and R 3.4.1 (R Project for Statistical Computing, Vienna, Austria).

## 3. Results

**Figure 1:**
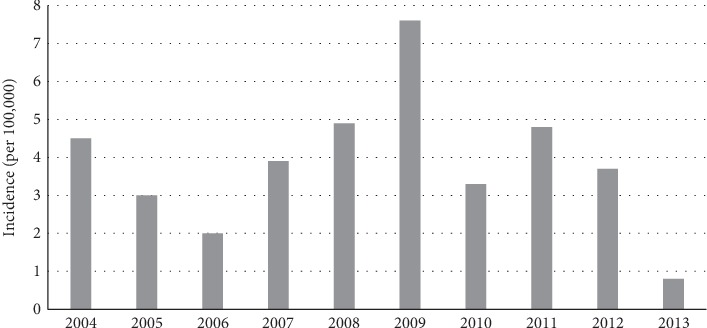
Incidence of keratoconus from 2004 to 2013 in Korea.

The number of patients diagnosed with keratoconus from 2004 to 2013 came to 1,552, and 7,760 controls were included for comparison. The incident cases were 46 (4.5%), 30 (3.0%), 20 (2.0%), 40 (3.9%), 49 (4.9%), 76 (7.6%), 33 (3.3%), 48 (4.8%), 37 (3.7%), and 8 (0.8%) in 2004, 2005, 2006, 2007, 2008, 2009, 2010, 2011, 2012, and 2013, respectively (Table 1 and Figure 1).

Table 2 shows the baseline characteristics of the study population. A total of 1,552 keratoconus patients and 7,760 subjects from the control group were included for analysis. There were no significant differences in demographic characteristics used for matching, including age group, sex, income, residential area, and the year of enrollment. Among the systemic diseases that were previously reported to be associated with keratoconus, allergic rhinitis (*p* < 0.001), asthma (*p* < 0.001), and atopic dermatitis (*p* < 0.001) were more prevalent in the keratoconus group, while DM was more prevalent in the comparison group (*p* < 0.001). Other diseases, including aortic dissection, sleep apnea, and collagen vascular diseases, did not show any significant difference between groups. Mitral valve prolapse and Down syndrome were excluded for further analysis because the prevalence in both groups was not measurable or very low.

Using multivariate logistic regression analysis, individuals with allergic diseases, including allergic rhinitis (OR: 1.86; 95% CI: 1.63–2.13; *p* < 0.001), asthma (OR: 1.20; 95% CI: 1.06–1.36; *p*=0.005), and atopic dermatitis (OR: 1.33; 95% CI: 1.13–1.56; *p* < 0.01), had significant associations with keratoconus after adjusting for confounding factors (Table 3). However, aortic dissection and sleep apnea did not show a significant association with higher or lower odds of keratoconus. Keratoconus also showed a significantly positive association with DM (OR: 1.35; 95% CI: 1.15–1.58; *p* < 0.001). No significant association was found for demographic factors because they were used for propensity matching.

## 4. Discussion

In the present study, the incidence rate of keratoconus ranged from 0.8 to 7.5 per 100,000 person-years during 2004 and 2013. After propensity score matching, logistic regression analysis showed significant associations with the presence of allergic rhinitis, asthma, and atopic dermatitis. However, no relevant result appeared in systemic diseases such as aortic dissection, sleep apnea, collagen vascular diseases, mitral valve prolapse, and Down syndrome.

Lee et al. [15] studied the epidemiology of keratoconus, which was designed in a similar manner but showed different results compared to our results. In contrast to our results, allergic conjunctivitis was the only risk factor for keratoconus in the Republic of Korea population in their study. Importantly, allergic rhinitis was not a risk factor for keratoconus, which differed from other studies, including our results. We suggest the difference in results was because their databases were from 2002 and 2015. They also used only age and household income for propensity scores, while in this study, we included age, sex, income, residential area, and year of enrollment to exclude any potential confounding factors that may affect the outcome. Additional studies are needed to confirm the results of various studies.

An epidemiological study from Germany reported that the incidence of keratoconus per 100,000 person-years was 13.3 (1 : 75,000), which showed a high incidence rate compared to our study [16]. The reason for this disparity may be due to the diagnostic codes, which may have included other conditions, as well as regional and racial differences among the patients included in the study. Another previously reported study in the Republic of Korea, which was a population-based study using the Korean Health Insurance Review and Assessment database, reported an incidence of keratoconus of 5.56 cases per 100,000 person-years [17], which was similar to the result from our study.

The conclusions reached in our study showed a significant association between allergic diseases including allergic rhinitis, asthma, atopic dermatitis, and the presence of keratoconus. An earlier study also reported that atopic diseases including asthma, vernal keratoconjunctivitis, atopic keratoconjunctivitis, and seasonal or perennial allergic keratoconjunctivitis were associated with keratoconus, and their prevalence in keratoconus patients had a higher incidence than the control group [18]. The most common cause of keratoconus is thought to be frequent eye rubbing, and pruritis associated with atopy and other allergic conditions leads to eye rubbing [19–24]. As a result of mechanical rubbing, friction occurs between the palpebral conjunctiva and corneal epithelium, which may eventually lead to corneal thinning, finally resulting in tissue bulging and a cone-shaped structure. Another possible theory suggests that the association between keratoconus and an allergic condition involves activation of the same human leukocyte antigens [19].

Importantly, our study showed a positive association between keratoconus and DM. Regarding the association between keratoconus and DM, there are several studies that have reported patient groups with DM showing lower odds of having keratoconus, when compared to groups without diabetics, with reduced odds ranging from 20% to 78% [10, 25, 27]. Studies reporting a low incidence of keratoconus in diabetics suggested that the results were due to thicker corneas in patients with diabetics. There are two hypotheses regarding the high stromal volume in diabetics. First, Ishino et al. [28] investigated corneal autofluorescence using a fluorophotometer and found that corneal autofluorescence in diabetics was higher than that of controls. They suggested mitochondrial dysfunction, water retention in the stroma, and increased corneal volume after observing the corneal autofluorescence. Another hypothesis suggested that high glucose levels stimulate nonenzymatic glycosylation, which leads to collagen cross-linking, stromal strengthening, and the prevention of age-related thinning [28]. However, Kosker et al. reported contradictory results in their case-control study, showing that patients with DM were more likely to suffer from keratoconus compared to normal controls [12]. The results of the present study are consistent with those of Kosker et al., showing a significant positive association between keratoconus and DM. However, further studies with a similar design are needed to confirm these results.

We also investigated the association between keratoconus and other systemic comorbidities, which were previously reported to be related with the disease. Previous studies have reported higher odds of keratoconus in patients with Down syndrome, mitral valve prolapse, collagen vascular disease, and sleep apnea [28–30]. However, the number of patients with these conditions was very low or absent in the cohort used for the analysis, so associations could not always be determined. Further studies with more patients are needed, therefore, to confirm the results.

The present study, based on reliable and numerous population groups, supported the positive correlation of keratoconus and allergic diseases. This study is one of the few studies that has investigated the epidemiology of keratoconus in the Korean population. However, there were some limitations to our study. The prevalence of keratoconus was lower than expected in some diseases, especially in mitral valve prolapse and Down syndrome, so the necessity of better-designated control groups is indicated. Moreover, it suggests the need for further investigations regarding other parameters, such as eye rubbing or sleep positions.

There are several limitations to consider when interpreting the results of our study. First, the diagnosis of keratoconus was entirely based on the KCD code system which may be inaccurate compared to the diagnosis made with a standardized diagnostic measurement. To overcome such limitations, we included those who had diagnostic code for keratoconus and underwent keratometry measurement, which may enhance the specificity of the keratoconus subjects included in this study. Second, it is possible that the OAG patients may have been underestimated because the data are based on hospital visits. The data do not include initial patients who did not experience symptoms and patients who do not visit the hospital for economic reasons. However, the degree of underestimation is limited due to the low cost and high accessibility of the Korean medical system, a government-run healthcare system including nearly the entire population of South Korea. Therefore, it is unlikely that inaccuracy and underestimation of the diagnosis affected the statistical results. Third, our data include only data for the Korean population, and there may be differences in other ethnic groups.

In summary, statistical analysis showed a significant association between keratoconus and atopic diseases such as allergic rhinitis, asthma, and atopic dermatitis. Considering the incidence of keratoconus with the above comorbid conditions, studies investigating this association may help in the planning of eye-care policies. However, conflicting results regarding the possible association between keratoconus and DM should be further evaluated.

## Figures and Tables

**Table 1:** Estimated incidence rate (per 100,000 person-years) of keratoconus in South Korea during 2004–2013.

Year	2004	2005	2006	2007	2008	2009	2010	2011	2012	2013
Population	1,016,391	1,016,646	1,001,891	1,020,593	1,000,601	998,348	1,001,898	1,006,332	1,011,013	1,014,622

Incidence	46	30	20	40	49	76	33	48	37	8

(per 100,000)	4.5	3.0	2.0	3.9	4.9	7.6	3.3	4.8	3.7	0.8

**Table 2:** Characteristics of study population.

Total	Keratoconus patients (*N*, %)		Matched control (*N*, %)		Total	*p* value
	1,552	100.0%	7,760	100.0%	9,312	
Allergic rhinitis						
No	342	22.0	2,810	36.2	3,152	<0.001
Yes	1,210	78.0	4,950	63.8	6,160	

Asthma						
No	1,048	67.5	5,833	75.2	6,881	<0.001
Yes	504	32.5	1,927	24.8	2,431	

Atopic dermatitis						
No	1,296	83.5	6,858	88.4	8,154	<0.001
Yes	256	16.5	902	11.6	1,158	

Aortic dissection						
No	1,550	99.9	7,753	99.9	9,303	0.655
Yes	2	0.1	7	0.1	9	

Sleep apnea						
No	1,548	99.7	7,746	99.8	9,294	0.527
Yes	4	0.3	14	0.2	18	

Collagen vascular diseases						
No	1,550	99.9	7,754	99.9	9,304	0.527
Yes	2	0.1	6	0.1	8	

Diabetes mellitus						
No	1,254	80.8	6,636	85.5	7,890	<0.001
Yes	298	19.2	1,124	14.5	1,422	

Mitral valve prolapse						
No	1,552	100.0	7,757	100.0	9,309	0.439
Yes	—	0.0	3	0.0	3	

Down syndrome						
No	1,552	100.0	7,759	100.0	9,311	0.655
Yes	—	0.0	1	0.0	1	

Variables for matching						

Sex						
Male	678	43.7	3,390	43.7	4,068	1.000
Female	874	56.3	4,370	56.3	5,244	

Age group						
≤19	233	15.0	1,165	15.0	1,398	1.000
20–59	1,045	67.3	5,225	67.3	6,270	
≥60	274	17.7	1,370	17.7	1,644	

Income						
Low	302	19.5	1,510	19.5	1,812	1.000
Middle	554	35.7	2,770	35.7	3,324	
High	696	44.8	3,480	44.8	4,176	

Residential area						
Metropolitan	130	8.4	650	8.4	780	1.000
City	401	25.8	2,005	25.8	2,406	
Rural	1,021	65.8	5,105	65.8	6,126	

**Table 3:** Logistic regression model estimating associations of covariates with keratoconus.

Variables		Odds ratio (95% CI)	*p* value
Allergic rhinitis	No	1.000 (reference)	
	Yes	1.86 (1.63–2.13)	<0.001

Asthma	No	1.000 (reference)	
	Yes	1.20 (1.06–1.36)	0.005

Atopic dermatitis	No	1.000 (reference)	
	Yes	1.33 (1.13–1.56)	<0.001

Aortic dissection	No	1.000 (reference)	
	Yes	1.17 (0.24–5.75)	0.853

Sleep apnea	No	1.000 (reference)	
	Yes	1.28 (0.42–3.92)	0.675

Collagen vascular diseases	No	1.000 (reference)	
	Yes	1.31 (0.26–6.65)	0.751

Diabetes mellitus	No	1.000 (reference)	
	Yes	1.35 (1.15–1.58)	<0.001

Variables for matching			

Sex	Male	1.000 (reference)	
	Female	0.92 (0.82–1.03)	0.135

Age group	≤19	1.000 (reference)	
	20–59	1.15 (0.97–1.36)	0.092
	≥60	1.01 (0.81–1.26)	0.495

Income	Low	1.000 (reference)	
	Middle	0.98 (0.84–1.15)	0.831
	High	0.94 (0.80–1.09)	0.337

Residential area	Metropolitan	1.000 (reference)	
	City	0.95 (0.77–1.19)	0.756
	Rural	0.96 (0.78–1.17)	0.744

## Data Availability

The Korean National Health Insurance Service National Sample Cohort database used to support the findings of this study were supplied by Korean National Health Insurance Service under license and so cannot be made freely available. Requests for access to these data should be made at the Internet homepage of Korean National Health Insurance Service (https://nhiss.nhis.or.kr).
